# Automating the PathLinker app for Cytoscape

**DOI:** 10.12688/f1000research.14616.1

**Published:** 2018-06-12

**Authors:** Li Jun Huang, Jeffrey N. Law, T. M. Murali

**Affiliations:** 1 Department of Computer Science, Virginia Tech, Blacksburg, VA, 24061, USA; 2 Genetics, Bioinformatics, and Computational Biology Ph.D. program, Virginia Tech, Blacksburg, VA, 24061, USA; 3 ICTAS Center for Systems Biology of Engineered Tissues, Virginia Tech, Blacksburg, VA, 24061, USA

**Keywords:** Network Biology, Shortest Paths, Pathway Reconstruction, CyREST API

## Abstract

PathLinker is a graph-theoretic algorithm originally developed to reconstruct the interactions in a signaling pathway of interest. It efficiently computes multiple short paths within a background protein interaction network from the receptors to transcription factors (TFs) in a pathway. Since December 2015, PathLinker has been available as an app for Cytoscape. This paper describes how we automated the app to use the CyRest infrastructure and how users can incorporate PathLinker into their software pipelines.

## Introduction

P athL inker is an algorithm that automates the reconstruction of any human signaling pathway by connecting the receptors and transcription factors (TFs) in that pathway through a physical and regulatory interaction network ^1^. In a comprehensive quantitative evaluation on NetPath pathways, P athL inker achieved higher reconstruction accuracy than several other state-of-the-art algorithms. In addition, P athL inker’s novel prediction that the cystic fibrosis transmembrane conductance regulator (CFTR), an ion-channel receptor, is involved in the Wnt pathway was experimentally validated ^1^. In general, P athL inker can be used to connect any set of sources to any set of targets in a given network.

The P athL inker app for Cytoscape is an implementation of this algorithm. The P athL inker app ^2^ was first released in the middle of December 2015. While it is possible to use the P athL inker app in conjunction with other Cytoscape apps, it can be cumbersome to create such workflows using the Cytoscape user interface. It is also challenging to reproduce the results of these workflows.

Here we present an CyREST-based API that allows users to incorporate P athL inker algorithms into their own software pipelines. The P athL inker application programming interface (API) will facilitate automated analysis of complex networks in reproducible workflows, including in conjunction with other CyREST enabled Cytoscape apps ^3^.

## Methods

### Implementation

The P athL inker API allows external software (written in languages such as Python and R) and tools (e.g., Jupyter Notebook) to access P athL inker functions via the REST protocol. The API follows the OSGi design pattern. The P athL inker API exposes two functions via JAX-RS annotations that allows them to be discovered by CyREST ^3^ and be made available to external callers via REST. We have documented these functions using Swagger annotations to meet the Cytoscape Automation documentation standards. The Swagger annotation allows the P athL inker API functions to be accessed via the Swagger user interface along with other API functions exposed by CyREST.

We substantially updated and refactored the P athL inker codebase to follow the principles of the OSGi modular design and to remove redundant code. Specifically, we refactored the code for generating the *k* shortest paths, network visualization, and functions related to the user interface (e.g., generating information in the “Result Panel”) into distinct Task classes that can be managed by the Task Manager in the Cytoscape API. The current design enables us to use the same codebase for running P athL inker through the Cytoscape user-interface as well as through the REST API. This modular design will facilitate easy expansion of the app in the future, e.g., by implementing additional subnetwork-finding algorithms.

### Operation

The P athL inker API is accessible directly through the Swagger user interface within Cytoscape or by using any REST-enabled client. Note that users must have installed v1.4 of the P athL inker app and Cytoscape v3.6.0 or higher. Moreover, an instance of Cytoscape must be running on the user’s computer. In the “Use Cases” section, we describe a sample workflow using py2cytoscape and provide an example Jupyter Notebook.

As shown in Figure 2, the P athL inker API provides two POST functions, “/pathlinker/v1/currentView/run” and “/pathlinker/v1/networkSUID/run”, which run P athL inker on the currently selected network and on the given networkSUID respectively. Both functions send user-selected source nodes and target nodes and a set of parameters to the P athL inker Cytoscape app.

The app computes the *k* shortest simple (loopless) paths that connect any source to any target in the network specified in the POST function, generates a subnetwork that contains these paths and a view of this subnetwork, creates a table in the Result Panel that contains these paths (See Figure 3b), and adds a “path rank” column to the Edge Table that contains the rank of the first path in which each edge appears. The POST functions return the computed paths, the SUIDs of the subnetwork and subnetwork view, and the name of the “path rank” column created by the app.

We summarize the parameters of the API functions and their outputs below.

**API parameters**

**Figure 1.**
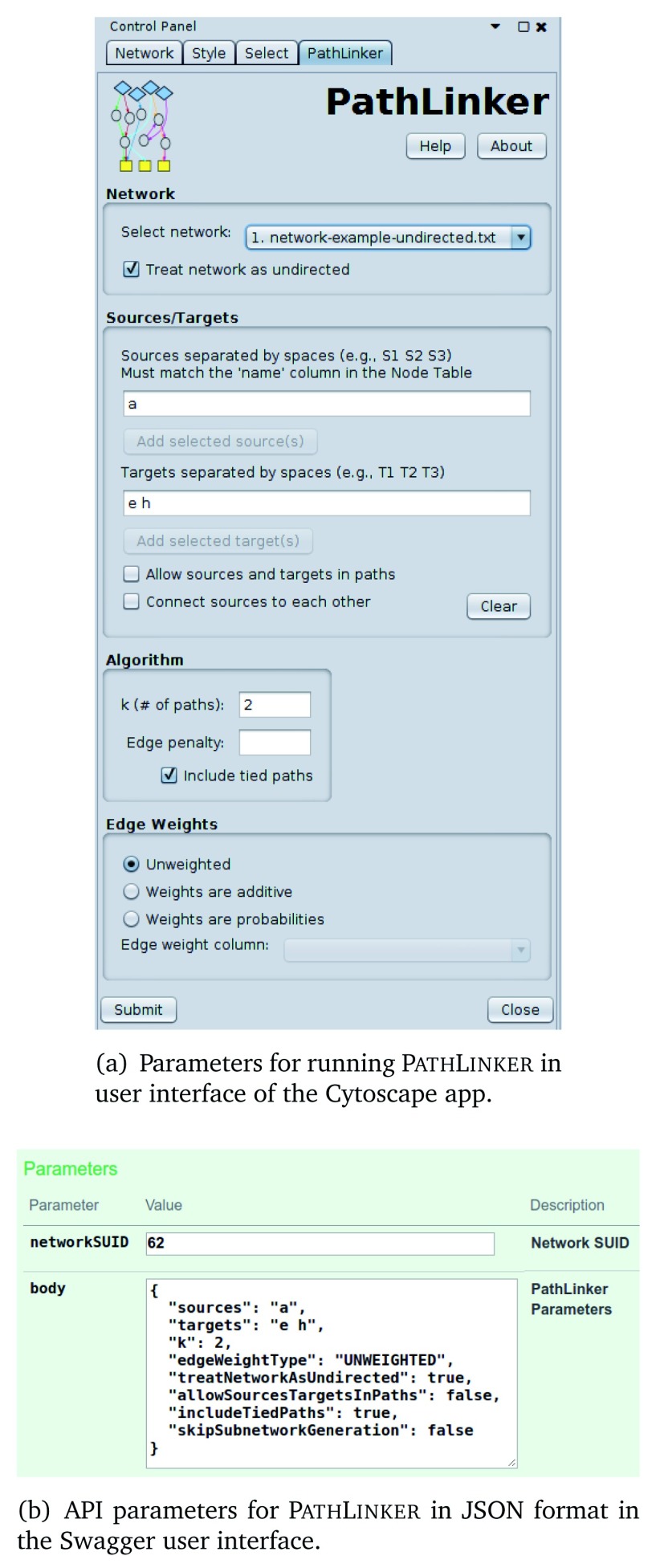
Comparison of P athL inker parameters in the app and in the API.

The API functions have the same set of parameters as the user can set in the user interface of the P athL inker app (see Figure 1a and Figure 1b). The user should provide these parameters in JSON format, whether they use the Swagger user interface or invoke the P athL inker API in code or via external tools. Table 1 contains an overview of the parameters, their types and a brief description. For a detailed description of the parameters, please see the Swagger documentation or documentation of the P athL inker app.

**Figure 2.**
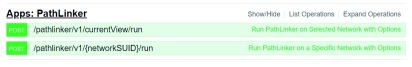
PathLinker application programming interface (API) functions shown in the Swagger user interface.

**Figure 3.**
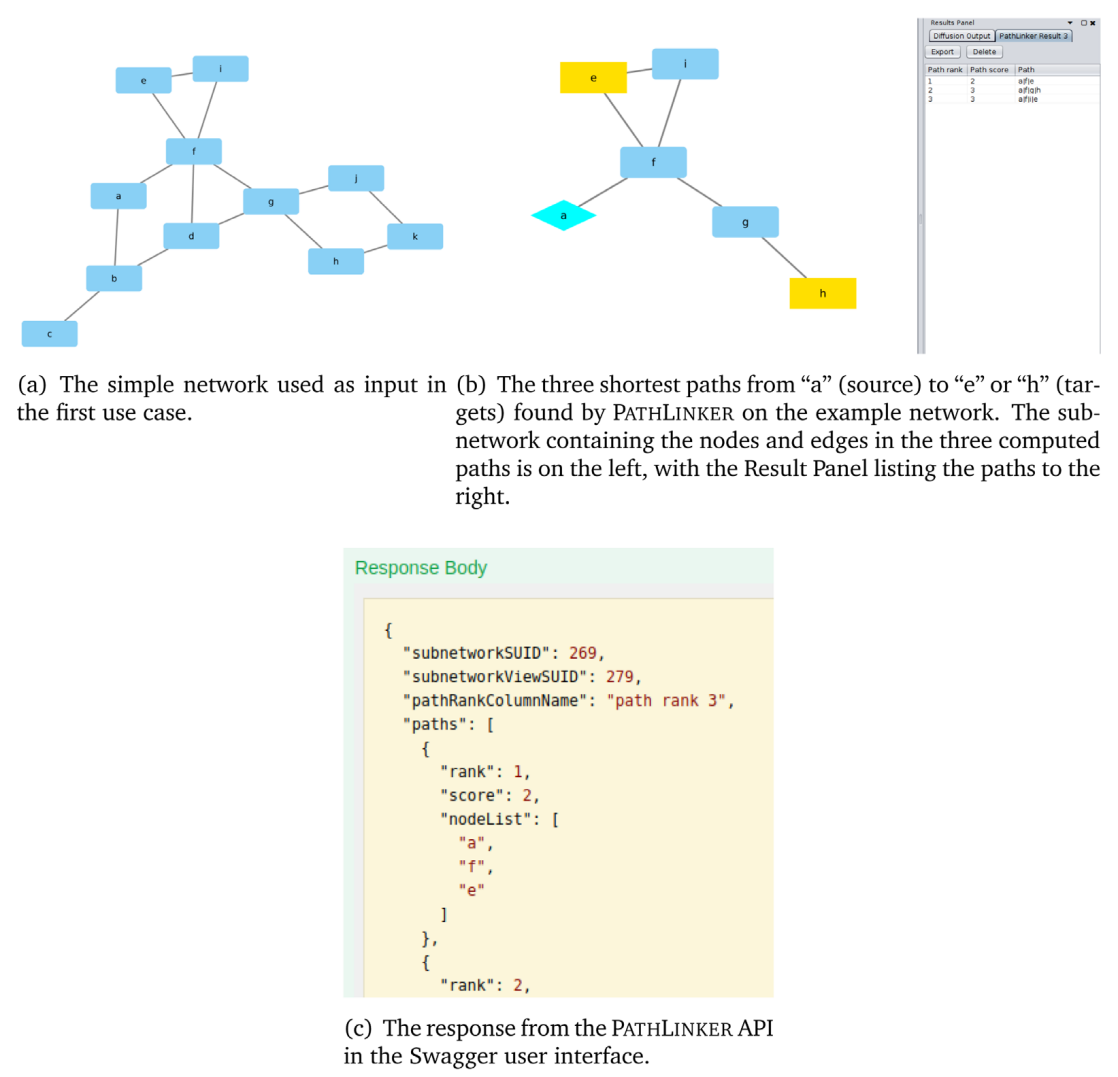
Illustration of the first use case.

**Table 1.** P athL inker application programming interface (API) parameters. The last column records whether an equivalent option is present in the user interface of the P athL inker app. T/F - True/False.

Parameter	Type	Description	Present in UI
sources	String	Node names separated by spaces	✓
targets	String	Node names separated by spaces	✓
*k*	Integer	Number of paths to compute	✓
edgeWeightType	String	UNWEIGHTED, ADDITIVE, or PROBABILITIES	✓
edgePenalty	Integer	Penalize low-weight paths with many edges	✓
edgeWeightColumnName	String	Name of the Edge Table column that contains the edge weights	✓
allowSourcesTargetsInPaths	T/F	Allow sources and targets to be intermediate nodes in paths	✓
includeTiedPaths	T/F	Include more than *k* paths if their score equals the *k*th path’s score	✓
treatNetworkAsUndirected	T/F	Ignore directionality when computing paths	✓
skipSubnetworkGeneration	T/F	Return only the *k* shortest paths	

**API Output**

The P athL inker API functions returns a response in JSON format with the following fields:
**subnetworkSUID:** The SUID of the subnetwork created in Cytoscape.
**subnetworkViewSUID:** The SUID of the subnetwork view created in Cytoscape.
**pathRankColumnName:** The name of the edge column created in the subnetwork’s edge table containing the rank of the first path in which each edge appears. The user can programmatically access this table using the CyREST API.
**paths:** The list of paths generated by the algorithm sorted by the path rank. This list contains the same information as the result table in the “Result Panel” created by the P athL inker app. Each entry in the list contains the following fields that describe a path:
**rank:** The rank of the path. For example, “100” for the 100th shortest path.
**score:** The total weight of the edges in the path.
**nodeList:** The list of nodes in the path. Each element in this list appears in the “name” column in the node table of the network provided as input to P athL inker. If multiple paths have the same score, then we order them lexicographically by their node lists.
If the user sets skipSubnetworkGeneration to true in the input to the API functions, then the P athL inker app does not generate the subnetwork and its view. Therefore, the API output will not contain the first three attributes, i.e., subnetworkSUID, subnetworkViewSUID, and pathRankColumnName.
The functions in the P athL inker API implement careful checks of the input. The function return the following error codes to provide meaningful feedback to the user:
**400:** Invalid user input. The JSON response lists all faulty input along with a reason why the input is correct, allowing the user to correct all the errors at the same time.
**404:** Current network or network with the given SUID does not exist.
**422:** No paths found. This error message indicates that P athL inker could not find any path connecting the source(s) to the target(s) in the given network. This error can occur if every source is disconnected from every target.

## Use cases

We provide two Jupyter Notebooks that illustrate the P athL inker API. The first notebook implements a use case on a simple network with 11 nodes and 13 edges, shown in Figure 3a. The notebook shows how to use Python and the py2cytoscape library to 1) load this network into Cytoscape, 2) call the P athL inker API with a set of parameters ( Figure 1), 3) view the computed paths and subnetwork, and 4) save the paths and/or subnetwork image to a file. The API function call in the notebook asks P athL inker to find the two shortest paths connecting “a” to “e” or to “h” while treating the network as undirected. It also asks P athL inker to return more than two paths if their scores are tied with the second path.

Even though we set *k* = 2, P athL inker returned three paths; Figure 3b shows the subnetwork containing these paths. P athL inker did so because we used the “Include tied paths” option, and the second and third path both had a score of three (they contain three edges each). In this example, we used the “Treat network as undirected” option because even though the edges in the network were intended to be undirected, py2cytoscape treats networks imported from the Python NetworkX package as directed.

The second notebook implements a more complex example that we presented in the paper describing the P athL inker app ^2^. Here, we used P athL inker to compute and analyze a network of interactions connecting proteins that are perturbed by the drug Lovastatin. The P athL inker API functions now allow us to automate this use case enabling fast reproduction of the results, as shown in the Jupyter Notebook.

## Summary

The subnetwork returned by the P athL inker API is another network in the current Cytoscape session. Hence, this network is amenable for analysis by other CyREST APIs and/or Cytoscape apps. Some examples include calling the Diffusion app’s API on the subnetwork, or modifying the subnetwork using standard CyREST API calls such as adding visual styles to the subnetwork or calling different layout algorithms.

In the near future, we plan to provide users more freedom in determining which column in the Node Table should act as the input for source and target fields. We will also implement additional sub-network generation algorithms. We will implement these new features in parallel in the P athL inker app and in the REST API.

We hope that these additions will increase making P athL inker a method of choice in the network biology community.

## Data availability

*All data underlying the results are available as part of the article and no additional source data are required.*

## Software availability

Software available from: http://apps.cytoscape.org/apps/pathlinker

The Cytoscape app source code and sample Jupyter Notebooks are available at https://github.com/Murali-group/PathLinker-Cytoscape

Archived source code as at time of publication: http://dx.doi.org/10.5281/zenodo.1252308 ^4^

License: GNU General Public License version 3
