# Supplementary material for: Automating the PathLinker app for Cytoscape
Source: F1000Res. 2018 Jun 12;7:727. [Version 1] doi: 10.12688/f1000research.14616.1 (PMC6051191; doi:10.12688/f1000research.14616.1)
Supplement: Peer review history

# Peer review: Referee response for version 1

**DOI:** 10.5256/f1000research.15906.r34937

**Published:** 2018-07-17

**Authors:** Stefan Wuchty

**Affiliations:** 1 Department of Computer Science, Center for Computational Science, University of Miami, Coral Gables, FL, USA

The manuscript ' Automating the PathLinker app for Cytoscape' by Huang, law and Murali introduces an API solution of their PathLinker software that was previously published. Although the PathLinker algorithm was made available through a Cytoscape app it turned out that its usage through the Cytoscape framework was initially cumbersome. With the new solution the authors used the CyREST API that allows to tap the results of the PathLinger app when used in conjunction with Cytoscape to be incorporated in user specific pipelines. As such, the authors allow through their API to use a Cytoscape app and results thus obtained to link to methods outside the Cytoscape framework. In particular, API output can easily be tapped using python and R code as well as using the Junyper notebook. 

 The proposed API appears very useful for researchers who want to consider the advantages of the PathFinder app w/o being constrained by the Cytoscape framework. The current manuscript describes the basic steps to get a user going. As a small change I would describe the examples in a bit more detail to show a fully worked example. In particular, Fig. 3 goes in that direction but is a bit generic. Working a real example would help with the transparency of the underlying approach.

I have read this submission. I believe that I have an appropriate level of expertise to confirm that it is of an acceptable scientific standard.

# Peer review: Referee response for version 1

**DOI:** 10.5256/f1000research.15906.r34942

**Published:** 2018-06-25

**Authors:** Alexander Pico

**Affiliations:** 1 Gladstone Institutes, San Francisco, CA, USA

Identifying paths through complex networks is a common use case. The PathLinker app provides a nice approach via GUI or scripting. The methods and use cases are detailed and clear. The provided Python Notebook is especially handy.

 My only suggestions for future versions of this work would be to support table column-based specification of sources and targets (already mentioned in your summary) as well as an R Markdown version of your script. The RCy3 package should make it easy to translate as it is analogous to py2cytoscape.

I have read this submission. I believe that I have an appropriate level of expertise to confirm that it is of an acceptable scientific standard.
